# Recent Progress in Therapeutic Angiogenesis

**Published:** 2007-09

**Authors:** Hironori Nakagami, Ryuichi Morishita

**Affiliations:** 1 *Division of Gene Therapy Science, Osaka University Graduate School of Medicine, Suita 565-0871, Japan;*; 2 *Division of Clinical Gene Therapy, Osaka University Graduate School of Medicine, Suita 565-0871, Japan*

**Keywords:** angiogenesis, bone marrow mononuclear cell, endothelial progenitor cell, hepatocyte growth factor

## Abstract

Coronary artery disease and peripheral arterial disease are devastating status of acute vessel occlusion in diseased vessels that are already narrowed enough by atherosclerotic process. People are now focused on therapeutic angiogenesis against the ischemic diseases, to supply and growth of new vessels into the ischemic tissue. Recently, we and others performed autologous transplantation of bone marrow mononuclear cell or endothelial progenitor cell and gene therapy using hepatocyte growth factor (HGF) in clinical trials. Autologous implantation of bone marrow mononuclear cells or endothelial progenitor cells in ischemic limb or heart may induce blood flow and improve function in several pilot clinical studies. The regenerative potential of bone-marrow cells can be explained by any of four mechanisms, transdiffentiaion, cytokine-induced growth, stimulation of endogenous stem cells, and induction of cell fusion. Transdifferentiaion has been described by previous investigators and the concept has been called into question by recent experimental studies. In the pilot clinical study, similar improvements were seen in gene therapy using HGF in peripheral arterial diseases which might be more noninvasive. The clinical significance of the novel therapeutic approach might embrace the large number of patients with peripheral arterial diseases and with chronic coronary artery disease. And we need to figure out the strong angiogenic factor or specific differentiated progenitor cells for therapeutic angiogenesis in the study of bone marrow cell therapy. For the patients, we need to constitute a safe and effective strategy for achievement of therapeutic angiogenesis.

## INTRODUCTION

Coronary artery disease and cerebrovascular disease are devastating status of acute vessel occlusion in diseased vessels that are already narrowed enough by atherosclerotic process. These diseases are caused basically by ischemic damage of the target organ, for example acute myocardial infarction from coronary artery and ischemic stroke from intracerebral artery occlusion. The prevention of these cardiovascular diseases involves the meticulous use of multiple drugs such as aspirin and beta-blockers, but these drugs are not enough to regenerate the organ or tissue that has been already damaged by preceding vascular events. People are now focused on tissue regeneration or gene therapy, and one method to do so is the supply and growth of new vessels into the ischemic tissue. It is now well-known that blood vessels are not only a ‘passive conduit’ for passage of blood components and gas, but an ‘active participant’ of several physiological/pathological processes that we encounter in everyday life in the clinics. In this review, we picked up autologous transplantation of bone marrow mononuclear cell or endothelial progenitor cell and gene therapy using hepatocyte growth factor (HGF) as a recent topic of therapeutic angiogenesis.

## THERAPEUTIC POTENTIALS OF BONE MARROW CELLS OR PROGENITOR CELLS TRANSPLANTATION

The regenerative potential of stem or progenitor cells is presently under intense investigation. They possess the capability of self-renewal and differentiation into organ-specific cell types, and transplantation of these cells may reconstitute organ systems. Human Endothelial Progenitor Cells (EPCs) have been isolated from the peripheral blood of adult individuals, expanded *in vitro*, and committed into an endothelial lineage in culture (1). The transplantation of these human EPCs has been shown to facilitate successful salvage of ischemic limbs of nude mice (2). These experimental findings call into question certain fundamental concepts regarding blood vessel growth and development in adult organisms. Postneonatal revascularization was considered synonymous with proliferation and migration of EC, angiogenesis (3) and homing of EPC to site of neovascularization, vasculogenesis (4) although the proportional contributions of angiogenesis and vasculogenesis remain to be clarified. Asahara *et al*. considered a novel strategy of EPC transplantation to provide a source of robust EC that might supplement fully differentiated EC thought to migrate and proliferate from preexisting blood vessels according to the classic paragram of angiogenesis developed by Folkman.

Tateishi-Yuyama and Matsubara *et al*. investigated efficacy and safety of autologous implantation of bone marrow-mononuclear cells in patients with ischemic limbs because of peripheral arterial disease (5). They injected bone marrow-mononuclear cells into gastronomies of the ischemic limb [ABI (ankle-brachial pressure index) < 0.6] in 45 patients. ABI in legs injected with bone marrow-mononuclear cells increased from 0.35 (95% CI 0.31-0.39) at baseline to 0.42 (0.38-0.46), and TcO_2_ (transcutaneous oxygen pressure) was increased from 28 mmHg [25-31] at baseline to 46 mmHg [42-50]. Exercise performance was strikingly improved in all patients who were able to treadmill exercise, and pain-free walking time increased from 1-3 min (95% CI 1.1-1.5) at baseline to 3-6 min (2.9-4.3). Therapeutic benefit was shown by complete regression of rest pain in 22 patients, striking improvement of rest pain to pain scale +1 in 15 patients, and improvement of ischemic ulcers in sex to ten patients. Their results suggests that autologous implantation of bone marrow-mononuclear cells could be effective for achievement of therapeutic angiogenesis. Minamino *et al*. did autologous implantation of peripheral-blood mononuclear cells in ischemic limbs of patients (6). They observed a significant increase in ABI (>0.1), rest pain in legs was greatly reduced in patients, and substantial improvement of ischemic ulcers was seen in patients. These results suggest that implantation of peripheral-blood mononuclear cells was similar to that of bone-marrow mononuclear cells.

A number of results in experimental studies have shown that intramyocardial implantation of bone marrow cells induce neovascularization and improve heart function after myocardial infarction. Tse HF *et al*. implanted antologous mononuclear bone marrow cells into the ischemic myocardium of eight patients with severe ischemic heart diseases as guided by electromechanical mapping with a percutaneous catheter procedure (7). After 3 months follow-up, patients reported a reduction of anginal episodes and in number of nitroglycerin tablets whereas their other antianginal drugs remained unchanged. None of the patients had arrhythmia detected on a 24 hour Holter recording at 3 months. MRI showed no abnormal intramyocardial mass and the left ventricle ejection fraction (LVEF) was unchanged, but there was an improvement in target wall thickening and motion. Furthermore, there was a reduction in the percentage mass of hypoperfused myocardium at 3 months. They indicated that intramyocardial implantation of bone marrow cells is safe and feasible for treatment of patients with ischemic heart diseases. Brehm *et al* treated 18 patients with chronic myocardial infarction using intracoronary transplantation of autologous bone marrow cells (8). After 3 months, infarct size in the transplantation group was reduced by 30% and both global left ventricular ejection fraction and infarction wall-movement velocity were increased significantly. Furthermore, there was an 11% improvement in maximum oxygen uptake and 115% increase in regional 18 F-fluordeoxyglucose uptake into infracted tissue. These results show that functional and metabolic regeneration of infracted and chronically avital tissue can be achieved in human using transplantation of bone marrow cells.

In patients with hematological diseases, granulocyte-colony stimulating factor (G-CSF) is a well established stem-cell mobilizer for peripheral-blood stem-cell transplantation, in which CD34 is used as a marker of hematopoietic stem-cells. Egashira *et al*. modified the autologous cell implantation in patients with ischemic limbs (9). The patients were given 5 μg/kg granulocyte colony-stimulating factor (G-CSF) for 4 days subcutaneously, and mononuclear cell aphaeresis was done on days 4-6 in whom the CD34 ratio has increased to more than 0.1% of peripheral white cells. The CD34-enriched cells were injected at ischemic limb muscle with the patients. Kang HJ and Kim HS *et al*. examined the feasibility and efficacy of granulocyte-colony stimulating factor (G-CSF) therapy and subsequent intracoronary infusion of collected peripheral blood stem-cells in 27 patients (cell infusion; n=10, G-CSF alone; n=10, and control group; n=7) (10). G-CSF injection and intracoronary infusion of cells did not aggravate inflammation and ischemia during the periprocedual period. Exercise capacity, myocardial perfusion, and systolic function improved significantly in patients who received cell infusion. However, they noted an enexpectedly high rate of in-stent restenosis in patients who received G-CSF. It could be a serious problem, and patients with G-CSF based stem-cell therapy should be carefully monitored for enexpected effects.

## HEPATOCYTE GROWTH FACTOR IN CARDIOVASCULAR SYSTEM

Hepatocyte growth factor (HGF) is a mesenchyme-derived pleiotropic factor that regulates growth, motility and morphogenesis of various types of cells, and is thus considered a humoral mediator of the epithelial-mesenchymal interactions responsible for morphogenic tissue interactions during embryonic development and organogenesis (11). Although HGF was originally identified as a potent mitogen for hepatocytes, the mitogenic action of HGF on human endothelial cells was most potent among growth factors (12). Moreover, the presence of a local HGF system (HGF and its specific receptor, c-met) was observed in vascular cells and cardiac myocytes *in vitro* as well as *in vivo* (13). We also demonstrated that HGF stimulated cell proliferation through the ERK-STAT3 pathway and had an anti-apoptotic action through the PI3K-Akt pathway in human aortic endothelial cells (14).

Furthermore, we have confirmed that intra-arterial administration of recombinant HGF induced angiogenesis in a rabbit hindlimb ischemia model (15) and examined the feasibility of gene therapy using HGF to treat peripheral arterial disease rather than recombinant therapy, because of its disadvantages. Intramuscular injection of 'naked' human HGF plasmid resulted in a significant increase in blood flow as assessed by laser Doppler imaging, accompanied by the detection of human HGF protein and a significant increase in capillary density. Importantly, at 5 weeks after transfection, the degree of angiogenesis induced by transfection of HGF plasmid was significantly greater than that caused by a single injection of recombinant HGF. As a preclinical study of human gene therapy, intramuscular injection of HGF plasmid once on day 10 after surgery produced significant augmentation of collateral vessel development on day 30 in a rabbit hindlimb ischemia model, as assessed by angiography. Serial angiograms revealed progressive linear extension of collateral arteries from the origin stem artery to the distal point of the reconstituted parent vessel in HGF-transfected animals. In addition, a significant increase in blood flow, assessed by a Doppler flow wire and the ratio of blood pressure in the ischemic limb to that in the normal limb, was observed in rabbits transfected with HGF plasmid (16).

## CLINICAL TRIAL OF THERAPEUTIC ANGIOGENESIS USING HGF

Therapeutic angiogenesis using angiogenic growth factors is expected to be a new effective treatment for patients with critical limb ischemia. We investigated the safety and efficacy of HGF plasmid DNA in patients with critical limb ischemia in a prospective open-labeled clinical trial (17). Patients could be enrolled if they (a) had had chronic critical limb ischemia, including rest pain or a non-healing ischemic ulcer, for a minimum 4 weeks; (b) had been resistant to conventional drug therapy for at least 4 weeks after hospitalization; (c) were not candidates for surgical or percutaneous revascularization based on usual practice standards; (d) did not have cancer or a history of cancer; and (e) did not have severe unstable retinopathy. Objective documentation of ischemia, including a resting ankle brachial index (ABI) of less that 0.6 in the affected limb on two consecutive examinations performed one week apart, was necessary. Patients were observed for 4 weeks under conventional drug therapy to confirm that their clinical symptoms and objective parameters were not improved. The selection criteria were confirmed by an independent committee for assessment and evaluation of clinical gene therapy at Osaka University, which was approved by the Ministry of Welfare and the Ministry of Education (Science and Culture).

Intramuscular injection of naked plasmid DNA was performed in the ischemic limbs of 6 patients with critical limb ischemia with arteriosclerosis obliterans (n=3) or Buerger disease (n=3) graded as Fontaine III or IV. First, a test intramuscular injection of a small dose (0.4 mg plasmid DNA) was performed to examine for acute or sub acute allergy to plasmid DNA. After confirmation of no allergic reaction or anaphylaxis, a therapeutic dose (2 mg) of naked HGF plasmid DNA was intramuscularly injected 2 weeks after the test injection. Four injection sites were selected, according to the angiographic findings and the available muscle mass. Four weeks after the initial injection, a second injection (2 mg) was similarly administered, giving a total dose of 4 mg plasmid DNA per patient.

The primary end points were side effects and improvement of ischemic symptoms at 12 weeks after transfection. For safety evaluation, we focused on the (a) allergic reaction against plasmid DNA, (b) the incidence of angiogenesis-related disease such as tumor, and (c) other severe complications. To identify an allergic reaction, we used a test injection of a small amount of plasmid DNA. However, neither the test, nor the initial or second therapeutic injection of human HGF plasmid DNA induced an allergic or anaphylactic reaction. Throughout the gene therapy period, there were no signs of systemic or local inflammatory reactions, and no critical side effects related to gene therapy were seen. To date, no development of tumors or progression of diabetic retinopathy has been observed in any patient transfected with HGF plasmid DNA during the trial. Two-month follow-up studies showed no evidence of development of neoplasm or hemangioma. In addition, no significant increase in serum HGF concentration was observed throughout the gene therapy period. We also measured the plasma level of plasmid HGF DNA. As expected, at 1 week after transfection, plasmid DNA could not be detected in the plasma, whereas at 1 day after transfection, a low level of plasmid DNA could be detected by polymerase chain reaction. Although one patient developed signs of cerebral infarction immediately after angiography during the trial period, the committee determined that this incident was related to the angiography catheter, and there was no relationship with the gene therapy. To date, no change in visual acuity has been observed in any patient treated with plasmid HGF gene transfer. It is noteworthy that no edema was observed in this trial, although transient lower-extremity edema was reported with clinical gene therapy using the VEGF gene, because of an increase in vascular permeability.

For efficacy evaluation, a reduction of pain scale of more than 1 cm on a visual analog pain scale was observed in 5 of 6 patients. An increase in ankle pressure index of more than 0.1 was observed in 5 of 5 patients. The long diameter of 8 of 11 ischemic ulcers in 4 patients was reduced by >25%. Thus, intramuscular injection of naked HGF plasmid is safe, feasible, and can achieve successful improvement of ischemic limbs. The efficacy of angiogenesis induction by plasmid DNA was also evaluated, although the patient number was small in this open-labeled trial. Unfortunately, it is difficult to detect distinct angiogenesis because angiography cannot visualize vessels <200 µm in diameter. Nevertheless, an improvement in digital subtraction angiography (DSA) findings was shown in 2 of 6 treated ischemic limbs. A large vessel was newly observed. Although it is not clear whether this vessel was new or not, it is possible that an increase of new microvessels led to recanalization. This recanalization was also confirmed by serial magnetic resonance angiograms. DSA in another patient with Buerger disease showed a marked increase in peripheral blood flow and formation of new blood vessels. To evaluate the functional improvement by HGF gene therapy, we also measured ABI (ankle brachial index) during gene therapy. Although ABI could not be measured in one patient because of uncompressible severely calcified vessels, ABI was significantly increased from 0.426 ± 0.046 (n=5) at baseline (before administration) to 0.626 ± 0.071 (*P*=0.0155; n=5) at 4 weeks after the second injection and to 0.596 ± 0.046 (*P*=0.0360; n=5) at 8 weeks after the second injection. The absolute value of systolic ankle pressure was significantly increased in 5 limbs after gene transfer, whereas ankle pressures of untreated limbs were not significantly changed. Also, TPI (toe pressure index), which could be measured only in 2 patients, tended to increase, accompanied by an improvement of ABI. However, TPI was not measured in 4 patients because of ischemic ulcers on the great toes of their ischemic legs. When an increase in ABI of >0.1 was assumed to be an improvement, according to the standard of Rutherford, 5 of 5 patients (100%) showed a positive response. In addition, as transcutaneous PO_2_ is an indicator of the effectiveness in terms of angiogenesis and increase in blood supply in targeted ischemic lesions, we also measured TcPO_2_. The change in TcPO_2_ after O_2_ stimulation was significantly increased at 8 weeks compared with baseline (*P*<0.05). To evaluate the effects of HGF gene therapy on clinical symptoms, we used the change in ischemic ulcers and visual analogue scale. In this trial, a total of 11 ischemic ulcers were found in 4 patients. Two of 11 ulcers completely disappeared. Considering an improvement of ischemic ulcers of more than 25% as positive, 8 of 11 ulcers (72%) improved. Three of 4 patients demonstrated an improvement of the ischemic ulcer longest diameter of >25% (efficacy rate=75%). Also, we evaluated resting pain using a visual analog scale, as a standard method for the evaluation of pain, where 0.0 cm means “pain free” or no pain, and 10 cm means most severe pain. Pain was significantly improved in a time-dependent manner.

Although the present data were obtained to demonstrate the safety in a phase I/early phase IIa trial, the initial clinical outcome with HGF gene transfer seems to indicate its usefulness as sole therapy for critical limb ischemia. Randomized placebo-controlled clinical trials of alternative dosing regimens of gene therapy will be required to define the efficacy of this therapy.

## FUTURE DIRECTION OF THERAPEUTIC ANGIOGENESIS

The clinical significance of the novel therapeutic approach might embrace the large number of patients with peripheral arterial diseases and with chronic coronary artery disease, preferably after previous myocardial infarction or a long-standing infraction. In terms of cell therapy, the regenerative potential of bone-marrow cells can be explained by any of four mechanisms:

- Direct cell differentiation from mononuclear cells to cardiac myocytes (18);
- Cytokine-induced growth and increase of residual viable myocytes, especially within the border zone of the infarcted area (19);
- Stimulation of intrinsic myocardial stem cells (endogenous stem cells) (20);
- Induction of cell fusion between transplanted bone marrow cells and resident myocytes (21).

Transdifferentiaion has been described by previous investigators and the concept has been called into question by recent experimental studies (22). The influence of cytokines restores coronary blood vessels and muscle cells after experimental myocardial infarction. Moreover, bone marrow cells contain a large quantity of cytokines (e.g. vascular endothelial growth factor, insulin-like growth factor, and platelet-derived growth factor), thereby stimulating residual normal myocytes for regeneration and proliferation and intrinsic myocardial stem cells (endogenous stem cells) for cell regeneration and cell fusion (23, 24). It is still unclear whether variation in the amount and kind of bone marrow cells, the administration technique, the transplantation procedure, the enhanced environment, and the improvement of the angiogenic micromilieu can enhance the milieu-dependent regenerative effect of stem cells in ischemic tissues. In terms of administration (e.g. intramuscular or intravascular), the injection of cells may be sometimes not mimicking strictly the natural course of the cells infused especially for the differentiation which can change the properties of these cells in terms of their capacity to incorporate into target tissue. In addition, although most experimental and clinical trials have shown early promise for cell transplantation into damaged hearts, most studies to date have analyzed ventricular function after a relatively short period of time. Recently, Kloner *et al* showed that neonatal cardiac cell transplantations exhibit long-term survival in a myocardial infarct model and contribute to long-term improved cardiac function, which suggest that a damaged heart can be rebuilt (25).

Gene Therapy using single gene may be more noninvasive than cell therapy. It might be very difficult to compare the efficacy of both therapies in this stage of clinical trial. Therefore, the long-term safety and treatment implications of gene therapy need to be investigated in double-blind studies with comparison group in large patient cohorts. Phase-III trial of HGF gene therapy for peripheral arterial diseases told us the answer for that.

**Figure 1:**
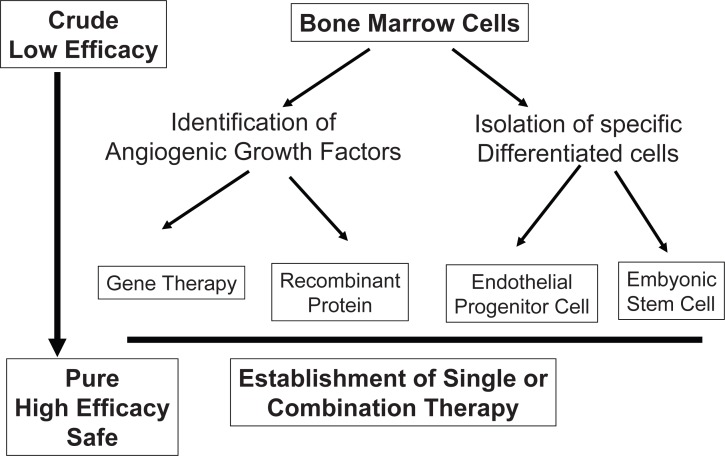
Trends in therapeutic angiogenesis: In the results of bone marrow cells transplantation, we need to figure out the strong angiogenic factor or specific differentiated progenitor cells for therapeutic angiogenesis. It may lead to achieve the safe and high efficient therapy.

The clinical significance of the novel therapeutic approach might enhance the large number of patients with ischemic disease. Now we have several clinical protocols for PAD or CAD, however we need to constitute a safe and effective strategy for achievement of therapeutic angiogenesis. As shown in Figure 1, we need to figure out the strong angiogenic factor or specific differentiated progenitor cells for therapeutic angiogenesis, and finally have to answer the question, “what is best therapy, mono therapy or combination therapy?” After the large clinical trial with well defined clinical protocol, we will be able to know the final decision based on these historical backgrounds.

## References

[1] Asahara T, Masuda H, Takahashi T, Kalka C (1999). Bone marrow origin of endothelial progenitor cells responsible for postnatal vasculogenesis in physiological and pathological neovascularization. Circ. Res.

[2] Kalka C, Masuda H, Takahashi T, Kalka-Moll W (2000). Transplantation of ex-vivo expanded endothelial progenitor cells for therapeutic neovascularization. Proc. Natl. Acad. Sci. USA.

[3] Folkman J (1971). Tumor angiogenesis: therapeutic implications. N. Eng. J. Med.

[4] Risau W (1995). Differentiation of endothelium. FASEB J.

[5] Tateishi-Yuyama E, Matsubara H, Murohara T, Ikeda U (2002). Therapeutic angiogenesis for patients with limb ischaemia by autologous transplantation of bone-marrow cells: a pilot study and a randomized controlled trial.

[6] Minamino T, Toko H, Tateno K, Nagai T (2002). Peripheral-blood or bone-marrow mononuclear cells for therapeutic angiogenesis?.

[7] Tse HF, Kwong YL, Cahn JKF, Lo G (2003). Angiogenesis in ischaemic myocardium by intramyocardial autologous bone marrow mononuclear cell implantation.

[8] Brehm M, Strauer BE (2006). Stem cell therapy in postinfarction chronic coronary heart disease. Nat. Clin. Pract. Cardiovasc. Med.

[9] Inaba S, Egashira K, Komori K (2002). Peripheral-blood or bone-marrow mononuclear cells for therapeutic angiogenesis?.

[10] Kang HJ, Kim HS, Zhang SY, Park KW (2004). Effects of intracoronary infusion of peripheral blood stem-cells mobilised with granulocyte-colony stimulating factor on left ventricular systolic function and restenosis after coronary stenting in myocardial infarction: the MAGIC cell randomised clinical trial.

[11] Nakamura T, Nishizawa T, Hagiya M (1989). Molecular cloning and expression of human hepatocyte growth factor. Nature.

[12] Van Belle E, Witzenbichler B, Chen D (1998). Potentiated angiogenic effect of scatter factor/hepatocyte growth factor via induction of vascular endothelial growth factor: the case for paracrine amplification of angiogenesis. Circulation.

[13] Nakamura Y, Morishita R, Higaki J (1995). Expression of local hepatocyte growth factor system in vascular tissues. Biochem. Biophys. Res. Commun.

[14] Nakagami H, Morishita R, Yamamoto K (2001). Mitogenic and anti-apoptotic actions of hepatocyte growth factor through ERK, STAT3, and AKT in endothelial cells. Hypertension.

[15] Morishita R, Nakamura S, Hayashi S (1999). Therapeutic angiogenesis induced by human recombinant hepatocyte growth factor in rabbit hind limb ischemia model as cytokine supplement therapy. Hypertension.

[16] Taniyama Y, Morishita R, Aoki M (2001). Therapeutic angiogenesis induced by human hepatocyte growth factor gene in rat and rabbit hindlimb ischemia models: preclinical study for treatment of peripheral arterial disease. Gene Ther.

[17] Morishita R, Aoki M, Hashiya N (2004). Safety evaluation of clinical gene therapy using hepatocyte growth factor to treat peripheral arterial disease. Hypertension.

[18] Beltrami AP, Barlucchi L, Torella D, Baker M (2003). Adult cardiac stem cells are multipotent and support myocardial regeneration. Cell.

[19] Orlic D, Kajstura J, Chimenti S, Limana F (2001). Mobilized bone marrow cells repair the infarcted heart, improving function and survival. Proc. Natl. Acad. Sci. USA.

[20] Leri A, Kajstura J, Anversa P (2002). Myocyte proliferation and ventricular remodeling. J. Card. Fail.

[21] Alvarez-Dolado M, Pardal R, Garcia-Verdugo JM, Fike JR (2003). Fusion of bone-marrow-derived cells with Purkinje neurons, cardiomyocytes and hepatocytes. Nature.

[22] Murry CE, Soonpaa MH, Reinecke H, Nakajima H (2004). Haematopoietic stem cells do not transdifferentiate into cardiac myocytes in myocardial infarcts. Nature.

[23] Torella D, Rota M, Nurzynska D, Musso E (2004). Cardiac stem cell and myocyte aging, heart failure, and insulin-like growth factor-1 overexpression. Circ. Res.

[24] Nadal-Ginard B, Kajstura J, Leri A, Anversa P (2003). Myocyte death, growth, and regeneration in cardiac hypertrophy and failure. Circ. Res.

[25] Muller-Ehmsen J, Peterson KL, Kedes L, Whittaker P (2002). Rebuilding a damaged heart. Circulation.

